# Effects of mobile apps on maternal weight-related and neonatal birth weight outcomes during pregnancy: a systematic review and meta-analysis

**DOI:** 10.1186/s12884-026-09778-8

**Published:** 2026-08-13

**Authors:** Andrea Rosa Paulini Bedon, Gian Carlos Saavedra Paredes, Carolina Delgado-Flores, Isabel Pinedo-Torres, Wendy Nieto-Gutierrez

**Affiliations:** 1 Universidad Científica del Sur, Lima, Perú; 2 Grupo Peruano de Investigación en Medicamentos, Políticas y Servicios Farmacéuticos (GPIMPSF), Carrera de Farmacia y Bioquímica, Universidad Científica del Sur, Lima, Perú; 3 Centro Cochrane y Grupo de Investigación NEMECS: Neurociencias, Metabolismo, Efectividad Clínica y Sanitaria, Universidad Científica del Sur, Lima, Perú; 4 Universidad San Ignacio de Loyola, Unidad de Investigación para la Generación y Síntesis de Evidencias en Salud, Lima, Peru

**Keywords:** Pregnancy, mobile applications, Gestational weight gain, Systematic review *(source: MeSH)*

## Abstract

**Background:**

Gestational weight gain outside recommended ranges is associated with adverse maternal and neonatal outcomes. Given their widespread use, mobile apps may support weight management during pregnancy.

**Aim:**

To evaluate the efficacy of mobile apps on maternal weight-related outcomes during pregnancy and neonatal birth weight-related outcomes.

**Methods:**

We conducted a systematic review and meta-analysis of randomized controlled trials evaluating mobile apps for weight management during pregnancy, with searches updated through July 2026. Comparable outcomes were pooled using fixed-effect and random-effects models, with random-effects estimates prioritized.

**Results:**

Twelve studies involving 3778 pregnant women were included. Mobile apps decrease the gestational weight (MD -0.67 kg; CI95% -1 to 0) and birth weight (MD -32 g; CI95% -140 to 75). However, a paradoxical trend was observed in which mobile apps appeared to reduce the risk of excessive maternal weight (RR 0.81; CI95% 0.66-1.00), while at the same time increased risk of inadequate maternal weight (RR 1.02; CI95% 0.67–1.54). This trend was also reflected in neonatal outcomes, as the risk of newborns being large for gestational age (RR 0.73; CI95% 0.54-1.00) and weight > 4000 g (RR 0.90; CI95% 0.74–1.09) appeared to decrease, whereas the risk of being small for gestational age (RR 1.22; CI95% 0.93–1.59) and birth weight < 2500 g (RR 1.41; CI95% 0.93–2.14) increased.

**Conclusions:**

Evidence on mobile apps for gestational weight management remains heterogeneous, suggesting potential benefits and harms. More precise evidence is needed to clarify clinical relevance and their implications for clinical practice.

**Clinical trial number:**

PROSPERO CRD42023458719.

**Supplementary Information:**

The online version contains supplementary material available at https://doi.org/10.1186/s12884-026-09778-8.

## Introduction

Maintaining adequate weight gain during pregnancy is essential for favorable pregnancy and delivery outcomes [1]. Nevertheless, it has been reported that only about 40% of pregnant women remain within the recommended weight limits, while the remaining 60% experience either insufficient or excessive gestational weight gain [2]. This is a concerning issue, as inadequate weight gain during pregnancy has been associated with adverse outcomes, delivery complications, and difficulties during breastfeeding [3], and is also closely related to the newborn’s weight which may consequently influence their nutritional status throughout life [4], increasing the risk of chronic diseases [5].

Currently, to improve maternal weight control, international organizations provide recommendations focused on lifestyle interventions (diet, exercise, etc.), maternal-fetal evaluation, and other related strategies [6, 7]. However, despite being part of routine prenatal care, these strategies have often proven insufficient for weight management during pregnancy [8, 9], considering adherence rates are frequently below 50% [10].

Mobile apps have emerged as potential tools in healthcare [11], with approximately 70% of the global population owning a mobile device and continued growth expected in the coming years [12]. In the context of pregnancy, new initiatives have encouraged the use of these technologies for monitoring, as they may improve adherence to and attendance at prenatal appointments, as well as facilitate the early detection of potential complications [13]. Although systematic reviews have examined the use of mobile apps as interventions to improve lifestyle [14, 15] and weight control in the general population [16], their efficacy in this field during pregnancy remains uncertain. This is particularly relevant given that pregnant women face unique challenges, such as physical limitations and contextual factors, which may hinder adherence to traditional strategies. In this scenario, mobile apps offer a viable and accessible alternative for gestational weight intervention, providing flexible follow-up that may help overcome common barriers. This study aimed to evaluate the efficacy of mobile apps with respect to maternal weight-related outcomes during pregnancy and neonatal birth weight outcomes.

## Methodology

### Study design and clinical question

This study is a systematic review of randomized controlled trials (RCTs). The review adhered to the Preferred Reporting Items for Systematic Reviews and Meta-Analyses (PRISMA) guideline [17] and followed the methodology outlined in the Cochrane Handbook for Systematic Reviews of Interventions [18]. The study protocol is registered in the PROSPERO database (Registration code: CRD42023458719). (Supplementary 1).

The clinical question was: “*What are the effects of mobile apps on maternal weight-related outcomes during pregnancy and neonatal birth weight-related outcomes?”*. This question was structured based on the following PICO components: [1] Population: pregnant women at any gestational age with a singleton pregnancy, given that weight control recommendations differ for multiple pregnancies; [2] Intervention: mobile apps, defined as programs (including text messaging) designed to operate on smartphones and tablets; [3] Comparator: standard gestational weight control without the use of mobile apps, defined as regular monitoring and management of maternal weight (nutritional and lifestyle recommendations) during prenatal care; and [4] Outcomes: gestational weight, birth weight, body fat, body mass index (BMI), and arm circumference.

### Search strategy

A systematic search of PubMed, the Cochrane Library, Embase, and Web of Science was conducted from database inception through 08 March 2023 and subsequently updated through 07 July 2026. Tailored search strategies were developed for each database using terms related to “mobile apps” and “gestational weight control.” No restrictions were applied to publication dates; however, only studies published in English, Spanish, or Portuguese were included. Additionally, reference lists of the included studies were screened to identify any additional eligible studies. The complete search strategies are provided in Supplementary 2.

### Study selection and data extraction

We included RCTs that addressed the clinical question. Studies were excluded if more than 50% of participants were pregnant women undergoing prolonged glucocorticoid treatment (over 10–15 days) or diagnosed with metabolic diseases (e.g., diabetes), because weight-management recommendations are individualized in these populations [19].

After removing duplicates using EndNote X20, two reviewers (ARPB and GCSP) independently and blindly selected studies through a two-step process, consisting of an initial screening of titles and abstracts followed by a full-text review. The selection process was conducted using the Rayyan QCRI web platform. Discrepancies were resolved through discussion with the involvement of a third reviewer (WNG).

A standardized data extraction table was developed to capture publication characteristics, study design, population, intervention and comparator details, and outcomes. Prior to data extraction, reviewers (ARPB, GCSP, and CDF) received training and then independently extracted the data. Disagreements were resolved by a third reviewer (WNG).

For birth weight and gestational weight, numeric values and categorical classifications were extracted. In the case of birth weight, categorical classification was based on World Health Organization definitions, [percentiles 10 and 90 for small and large for gestational age; and < 2500 g and > 4000 g thresholds [20, 21]]. For gestational weight, categorical classification was based on the Institute of Medicine criteria [22] [excessive weight gain: weight above the upper limit for the pre-pregnancy BMI category; and inadequate gain: below the lower limit].

### Risk of bias assessment and certainty of the evidence

Risk of bias was assessed using the RoB 2 tool [23], which considers the following domains: bias arising from the randomization process, deviations from intended interventions, missing outcome data, measurement of the outcome, and selection of the reported result. This assessment was performed independently and in duplicate by two reviewers (ARPB, GCSP, and CDF), with any discrepancies resolved by a third reviewer (WNG).

We assessed the certainty of the evidence for each outcome using the GRADE approach for intervention studies [24]. The certainty of the evidence was categorized as high, moderate, low, or very low; based on the risk of bias, imprecision, inconsistency, indirectness, and publication bias domains. Imprecision was evaluated using a partially contextualized approach [25]. Clinically important differences were predefined for each continuous outcome: every change of 71 g for birth weight [26], 1 kg/m² for BMI [27], 7 kg for gestational weight [28], 2% for body fat, and 2 cm for arm circumference [29]. For dichotomous outcomes, every 25% difference was considered clinically relevant.

### Statistical analyses

Data were analyzed using R software. Study characteristics were summarized descriptively. Where outcomes were sufficiently comparable across studies, results were synthesized using both fixed-effect and random-effects meta-analyses. Random-effects estimates were prioritized because clinical and methodological heterogeneity across studies was anticipated.

For dichotomous outcomes reported as event counts, risk ratios (RRs) were calculated from 2 × 2 tables and pooled using the *metabin* function in R. When adjusted effect estimates were reported, these were preferentially used because they accounted for relevant covariates. Adjusted RRs and their 95% confidence intervals (CIs) were transformed to the natural logarithmic scale and pooled using the generic inverse-variance method implemented with the *metagen* function in R. The pooled log RR and its 95% CI were then exponentiated and presented as RR with 95% CI. For studies in which RRs had to be estimated from 2 × 2 data before log transformation, a continuity correction of 0.5 was added to each cell only when zero events were reported [30]. For continuous outcomes reported as means and standard deviations, mean differences (MDs) were pooled using the *metacont* function in R. When adjusted estimates were available, these were pooled using the generic inverse-variance method.

For all random-effects meta-analyses, between-study variance was estimated using the Paule–Mandel method, and the confidence interval for τ² was estimated using the Q-profile method.

Subgroup analysis was not feasible due to the limited number of studies per outcome.

## Results

### Characteristics of studies included

**Fig. 1:**
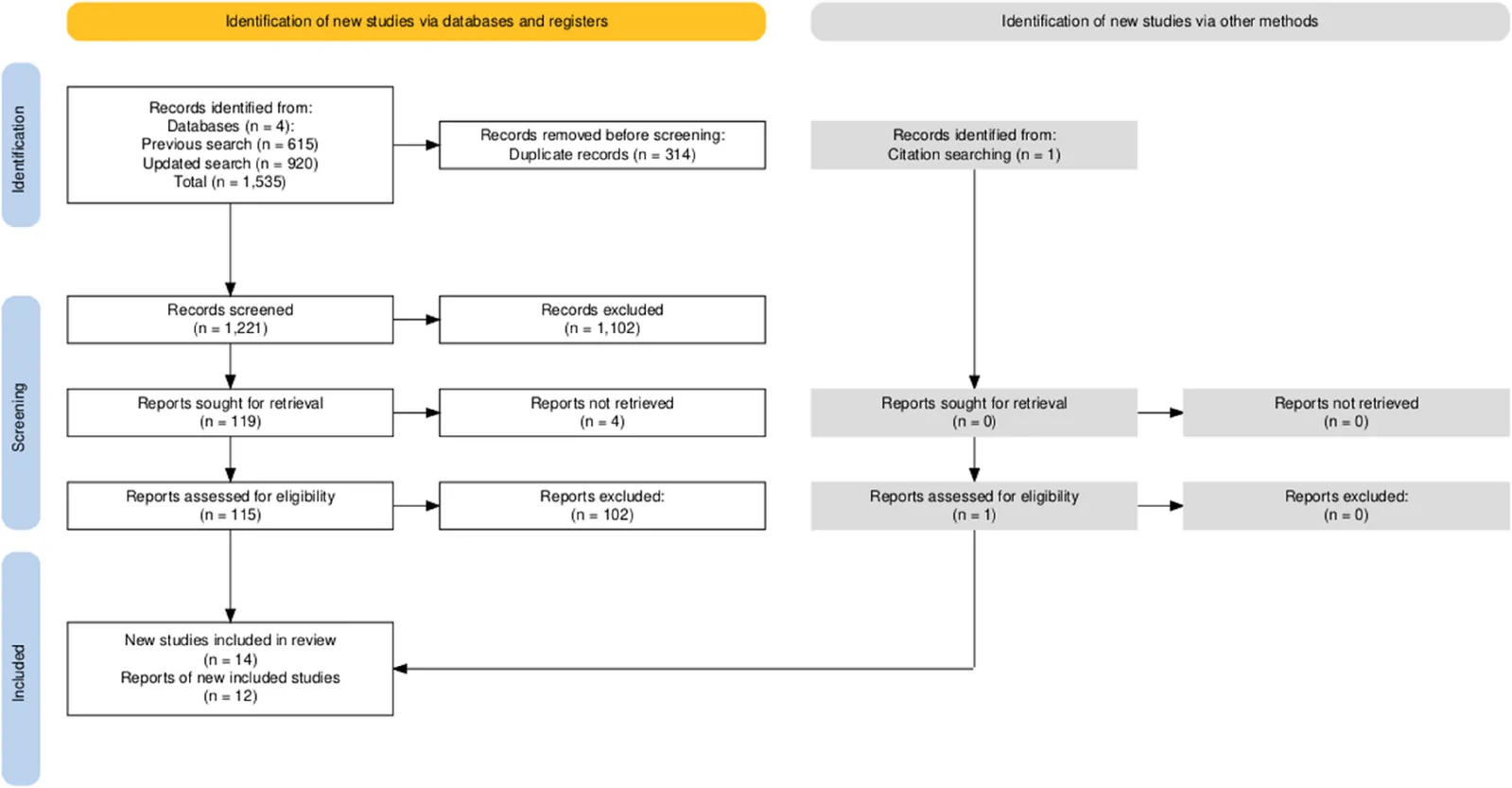
Flow diagram of study selection. The PRISMA flow diagram was created using the online tool available at https://estech.shinyapps.io/prisma_flowdiagram/. The previous search covered records up to March 2023, and the updated search covered records up to July 2026

A total of 1535 studies were identified. After removing duplicates, 1221 records were screened. Of these, 1102 were excluded based on titles and abstracts, and 115 full-text articles were assessed for eligibility. Thirteen reports were identified through the database searches, and one additional report was identified through reference-list screening, yielding 14 studies [11, 31–43] describing 12 unique RCTs. (Fig. 1). The list of the excluded studies and the reasons for exclusion are detailed in Supplementary 3.

The studies evaluated a total of 3,778 pregnant women aged between 18 and 45 years, from various countries, primarily in Europe and Asia. The studies evaluated heterogeneous mobile apps interventions, such as HealthyMoms, Glycoleap, MyHealthWeight, mHEMB, Better Pregnancy, and Adaptive LEAP, most of which were delivered in addition to standard maternity care. Several interventions incorporated additional components, such as wearable activity trackers, SMS or email reminders, behavioral weight-management training, educational sessions, remote monitoring, and individualized coaching or counseling. In two studies, text messaging was used as the primary intervention. All interventions aimed to monitor gestational weight gain, provide nutritional education, and promote physical activity. Intervention duration ranged from 4 weeks to 8 months, with some interventions continuing until delivery. Control groups received standard prenatal care, which included routine medical monitoring, general lifestyle counseling, and periodic maternal-fetal health assessment. (Table 1).

**Table 1:** Characteristics of the included studies

Author (year)	Design	Tracking time	Country	Population	Age - years Mean (SD)	% Nulliparous	Gestational age - weeks Mean (SD)	Weight - kg Mean (SD)	Intervention (*N*)	Device Description	Time of intervention use	Comparator (*N*)
Sandborg 2021 [31]	Simple blinded (participants and outcome assessors were not blinded), single-center RCT (restricted randomization, ratio 1:1), and parallel design	24 weeks. At 14 and 37 weeks of gestation, all outcomes were assessed	Sweeden	Incl: Pregnant women (18 + years, singleton pregnancy) with sufficient Swedish proficiency. Excl: Those with a history of eating disorders, diabetes, or weight-affecting medical conditions.	31.3 (4.1) I: 31.4 (4.3) C: 31.3 (3.8)	57.4 I: 56.6 C: 58.2	13.9 (0.7) I: 13.8 (0.6) C: 14.0 (0.7)	67.7 (11.5) I: 68.3 (12.8) C: 67.0 (10.2)	HealthyMoms app (Android and iOS compatible + standard maternity care (*N* = 152)	The app includes biweekly themes, push notifications, self-monitoring with feedback (diet, activity, GWG), recipes, an exercise guide with videos, a pregnancy calendar, and a library with FAQs and tips.	24 weeks (6 months)	Regular monitoring of maternal and fetal health (vitals, weight, fetal movements), an early pregnancy lecture on a healthy lifestyle, and medical care, including midwife visits and fetal diagnostics. (*N* = 153)
Kennelly 2018 [32]	Open label, single-center RCT (central randomization, ratio 1:1, stratified by BMI), and parallel design	At 28 and 34 weeks (only for weight measure) of gestation	Ireland	Incl: Pregnant women (BMI 25.0–39.9) with a singleton pregnancy, between 10–20 weeks of gestation, smartphone users, and fluent in English. Excl: Women with medical conditions requiring dietary restrictions.	I: 32.8 (4.6) C: 32.1 (4.2)	I: 49.6 C: 53.7	15.5	I: 79.5 (11.3) C: 78.6 (11)	“Healthy Lifestyle Package”: One educational session (at the beginning in of the study) + reinforced through smartphone application and email every 2 weeks (*N* = 278)	A database of low-glycemic recipes, an exercise advice section, and a homepage with daily nutrition and exercise tips	16.2 weeks (SD: 8)	Diet, exercise or weight gain tips (*N* = 287)
Li 2019 [11]	Open label, single-center RCT (ratio 1:1), and parallel design	At 22–24 weeks’ (4 w) and 26–28 weeks’ (8 w) gestation	Singapore	Incl: Pregnant Singapore citizens or residents (≥ 21 years, BMI ≥ 25) between 18–20 weeks’ gestation, fluent in English, and able to use a smartphone app. Excl: Women with medical conditions requiring dietary restrictions.	I: 29.3 (4.4) C: 30.7 (5)	I: 47 C: 33	I: 19.2 (2.1) C: 17.6 (2.8)	NR	Food-coaching smartphone app (Glycoleap, Holmusk, Singapore) (*N* = 15)	Upload food images to receive real-time dietitian feedback (8 AM–8 PM), including ratings (1–5) and guidance on food balance and composition.	NR	Basic nutrition principles for selecting nutrient-rich foods and limiting high-fat, high-sugar foods and drinks. (*N* = 15)
Gonzales-Plaza 2022 [34]	Single blinded, single-center RCT (central randomization, ratio 1:1), and parallel design	I: 21.5 (3.2) weeks C: 21.1 (2.4) weeks	Spain	Incl: Pregnant women (≥ 18 years, BMI ≥ 30) at 12–18 weeks of a singleton pregnancy, with internet access on an Android or iPhone, and willing to participate. Excl: Those already using an app for activity/weight tracking, diagnosed with psychiatric or endocrine-metabolic disorders, chronic hypertension, mobility issues preventing moderate walking, or difficulty understanding Spanish.	I: 32.4 (5.4), C: 33.4 (4.7)	I: 54 C: 39	12 to 18 weeks	I: 86.1 (10.4) C: 84.3 (9.9)	Standard maternity care + Smartband (Mi Band 2) + Mi Fit App + Hangouts App (*N* = 78)	Recommendation to take 10,000 steps daily (≥ 5 days/week) or 30 min of moderate activity, tracked via the Mi Fit app and Smartband. Midwife support is provided twice a week via Hangouts (SMS or video) on maternal and fetal changes, nutrition, weight, exercise, pregnancy, labor, and postpartum.	NR	Oral and written guidance on physical activity, recommending 30 min/day of moderate exercise (≥ 5 days/week), a GWG of 5–9 kg during pregnancy, and a balanced 1800 kcal Mediterranean diet. (*N* = 72)
Singh 2020 [33]	Open label, cluster (52 cluster) RCT (1:1), and parallel design	At 13–28 weeks to 38–39 or end of the pregnancy weeks and 7 months after delivery	Nepal	Pregnant women (15–45 years) in their second trimester (13–28 weeks).	<=19 years: 22.8 I: 20.1 C: 25.6	39.0 I: 32.7 C: 45.7	NR	45.80 (5.72) I: 45.93 (5.90) C: 45.66 (5.52)	Training + monitoring + text message (*N* = 214)	Training covered maternal and child health services and recommended maternal diets, followed by monthly supervision. Pregnant women received text messages on health services and dietary recommendations during pregnancy and postpartum.	Between the 4th and 6th months and weekly thereafter childbirth	Standard Control (NR) (*N* = 199)
Huang 2019 [35]	Open label, multi-center RCT (central randomization, ratio 1:1, stratified by BMI), and parallel design	2 weeks into the intervention and then at 28 weeks gestation or 36 weeks gestation, and 3 months post-delivery	Australia	Incl: Women (≥ 18 years, < 11 weeks gestation, BMI ≥ 20) fluent in conversational English. Excl: Those who conceived with donor eggs/sperm, had pre-existing medical conditions or were carrying multiples.	33.26 (4.16) I: 32.47 (4.18) C: 34.15 (4.02)	NR	9.21 (1.21) I: 9.06 (1.29) C: 9.38 (1.12)	I: 73.65 (67.49–79.81) C: 9.52 (64.48–74.56)	PLAN intervention + standard maternity care + text message (*N* = 30)	App web-based intervention providing IOM-based real-time weight feedback, diet and physical activity guidance, goal setting, self-monitoring tools, one personalized dietitian session, and weekly SMS support.	12 weeks	Standard Control (NR) (*N* = 27)
Herring 2019* [36]	Single blinded (Staff collecting), multi-center RCT (central randomization, ratio 1:1), and parallel design	6 months at 33–38 weeks of gestation and 6 months post-partum	USA	Incl: Pregnant women (≥ 18 years, < 22 weeks gestation, BMI 25.0–44.9) with a cell phone and unlimited texting. Excl: BMI ≥ 45, multiple pregnancies, prior bariatric surgery, chronic conditions affecting weight (e.g., diabetes, thyroid disorder, HIV, severe depression), or significant tobacco (> 5 cigarettes/day) or drug use.	25.6 (5.4) I: 26.0 (5.3) C: 25.3 (5.4)	38 I: 37 C: 39	13.9 (4.1) I: 13.7 (4.1) C: 14.1 (4.1)	NR	Multi-component: Behavioral weight control training + text message + counseling support (coach and Facebook) (*N* = 131)	Behavior changes goals supported by daily feedback texts, web-based weight gain graphs, health coaching, and a Facebook support group.	NR	Standard prenatal care included routine obstetric visits for maternal and fetal monitoring, counseling on nutrition and substance use, and, for most participants, WIC food vouchers and nutrition counseling during pregnancy. (*N* = 131)
Chen 2023 [38]	Blinded NR, multi-center RCT (central randomization, ratio 1:1), and parallel design	At < 17 weeks of gestation to 6 months postpartum	Taiwan	Incl: Pregnant women (≥ 20 years, < 17 weeks gestation, BMI ≥ 25) with fluent in Mandarin Chinese, and able to understand basic technologies. Excl: Diagnosis of an eating-related disorder, diabetes, or medical conditions that influenced body weight	32.8 (4.96)	47.8 I: 47.8 C: 47.8	13.9	74.8 (11.3) I: 74.4 (11.8) C: 75.1 (10.9)	MyHealthWeight (MHW) app + Mi Smart Band 5 + text message (SMS) (*N* = 46)	The app included features such as goal setting, progress charts, rewards, reminders, and prenatal tools. The smart band helped participants monitor physical activity.	20 weeks	Regular monitoring of maternal and fetal health, including body weight. Participants could also optionally receive brief healthy lifestyle counseling during early pregnancy, including general advice on diet. (*N* = 46)
Chen 2024 [37]												
Chen 2025 [40]	Open label, single-center RCT (1:1), and parallel design.	At < 17 weeks of gestation to 6–12 weeks postpartum	Taiwan	Incl: Singleton pregnancy (> 18 years, in the first 20 weeks, BMI ≥ 24), having a smartphone, and having the ability to communicate. Excl: Limited physical activity or the inability to perform exercise, pregnancy induced hypertension, or other obstetric complications.	I: 35.7 (4.3) C: 35.8 (4.3)	57.1 I: 62.5 C: 51.8	I: 16.4 (4.1) C: 15.8 (2.9)	I: 63.8 (15.4) C: 62.4 (13.6)	Standard maternity care + mHEMB + text message (SMS) (*N* = 56)	Included weekly self-monitoring of weight, diet, and exercise, tailored health education, goal setting, data trend feedback, reminders, incentives, and monthly coaching via video or SMS.	6–8 months	Standard clinic-based prenatal care, including routine pregnancy checkups and a maternal health booklet with guidance on prenatal weight gain, diet, exercise, birth preparation, and pregnancy-related lifestyle recommendations. (*N* = 56)
Chen 2025 (b) [39]												
Duan 2026 [41]	Single blinded (staff), multi-center RCT (central randomization, ratio 1:1), parallel design.	At < 12 weeks of gestation to delivery	China	Incl: Singleton pregnancy (18–49 years, < 12 weeks gestation), must use Android smartphones, have at least one risk factor for gestational diabetes mellitus, and have no heart, liver, or kidney diseases during pregnancy Excl: Pre-existing diabetes, gestational diabetes mellitus diagnosed in late pregnancy, threatened miscarriage, obstetric complications restricting physical activity, or concurrent participation in other research projects.	< 35 years: 57.3% ≥ 35 years: 42.7%	NR	NR	NR	Better Pregnancy (Android) + standard care (*N* = 122)	App included GDM risk prediction, personalized diet/exercise/weight plans, daily self-monitoring, automated reminders, tailored professional feedback, and family/peer support	NR	Standard prenatal care, including GDM risk screening, pregnancy risk classification, educational pamphlet/self-learning resources, online prenatal school, and routine monthly prenatal visits with blood pressure and weight monitoring before 28 weeks (*N* = 124)
Feng 2023 [42]	Open label, single-center RCT (1:1), and parallel design.	From 6–7 weeks until delivery	China	Incl: Singleton pregnancy (20–40 years, 6–7 weeks, BMI ≥ 24) with no previous history of childbirth, and start routine antenatal care from 12 gestational weeks Excl: Pre-pregnant diabetes, hypertension or other cardiovascular diseases, complications with systemic lupus erythematosus and other autoimmune diseases, multiple pregnancy, and preterm delivery.	I: Median 32.0 (IQR 30.0–35.0) C: 33.0 (31.0–36.0)	100	I: Median 39 (IQR 38–40) C: 39 (38–39)	I: Median 28.9 (IQR 27.6–20.9) C: 29.3 (27.8–31.2)	Smartphone app + plus usual care (*N* = 143)	Smartphone app–assisted gestational weight management, including personalized weekly weight-gain targets, calorie intake/expenditure goals, self-monitoring of weight, diet and physical activity, trend feedback, reminders, and pregnancy education.	29 weeks (IQR 23–32)	Usual antenatal care only, including standard trimester-specific weight-management education on diet, recommended GWG, exercise, nutrition status, GDM screening, and communication with obstetricians. (*N* = 138)
Hedderson 2026 [43]	Double blinded, cluster (58 cluster) RCT (1:1), and parallel design	From 8–15 weeks until delivery	USA	Incl: Singleton pregnancy (≥ 21 years, 8 to 15 weeks, BMI 25–29), English-speaking, had smartphone and Wi-Fi access.	33.4 (4.6) I: 33.3 (4.7) C: 33.5 (4.5)	41.6 I: 44.8 C: 37.9	10.3 (1.9) I: 10.3 (1.9) C: 10.2 (1.9)	78.7 (12.5) I: 78.2 (12.5) C: 79.3 (12.5)	Adaptive LEAP mHealth + standard care (*N* = 677)	App provided personalized automated feedback on gestational weight gain and physical activity, delivered weekly educational content, and supported self-monitoring of weight, diet, and activity through connected devices.	25.4 (5.6) weeks	Standard prenatal care alone, including routine prenatal visits, weight measurement at visits, and newsletter with IOM GWG guidelines and healthy eating tips. (*N* = 588)

*BMI* Body mass index, *C* Control group, *Exc* Excluded, *FAQs* Frequently asked question, *GWG* Gestational weight gain, *GI* Glycemic index, *Incl* Included, *I* Intervention group, *NR* Not referred, *RCT* Randomized controlled trial, *GDM* Gestational diabetes mellitus, *IQR* Interquartile range, *GWG* Gestational Weight Gain, *IOM* Institute of Medicine

*Does not report estimates for the effect

### Birth weight

Eight studies [32, 34, 35, 37, 40–43] reported birth weight outcomes, three using categorical classifications of percentiles (90th and 10th for large and small for gestational age, respectively), seven using weight cut-offs (> 4000 g and < 2500 g), and six reporting numerical values (g). Analyses based on birth-weight percentiles suggested that mobile app probably reduce the risk of delivering a large-for-gestational-age newborn (RR, 0.73; 95% CI, 0.54–1.00), corresponding to 17 fewer cases per 1,000 newborns (29 fewer to 0 fewer). Conversely, the interventions may increase the risk of delivering a small-for-gestational-age newborn (RR, 1.22; 95% CI, 0.93–1.59), corresponding to 8 more cases per 1,000 newborns (3 fewer to 22 more).

**Fig. 2:**
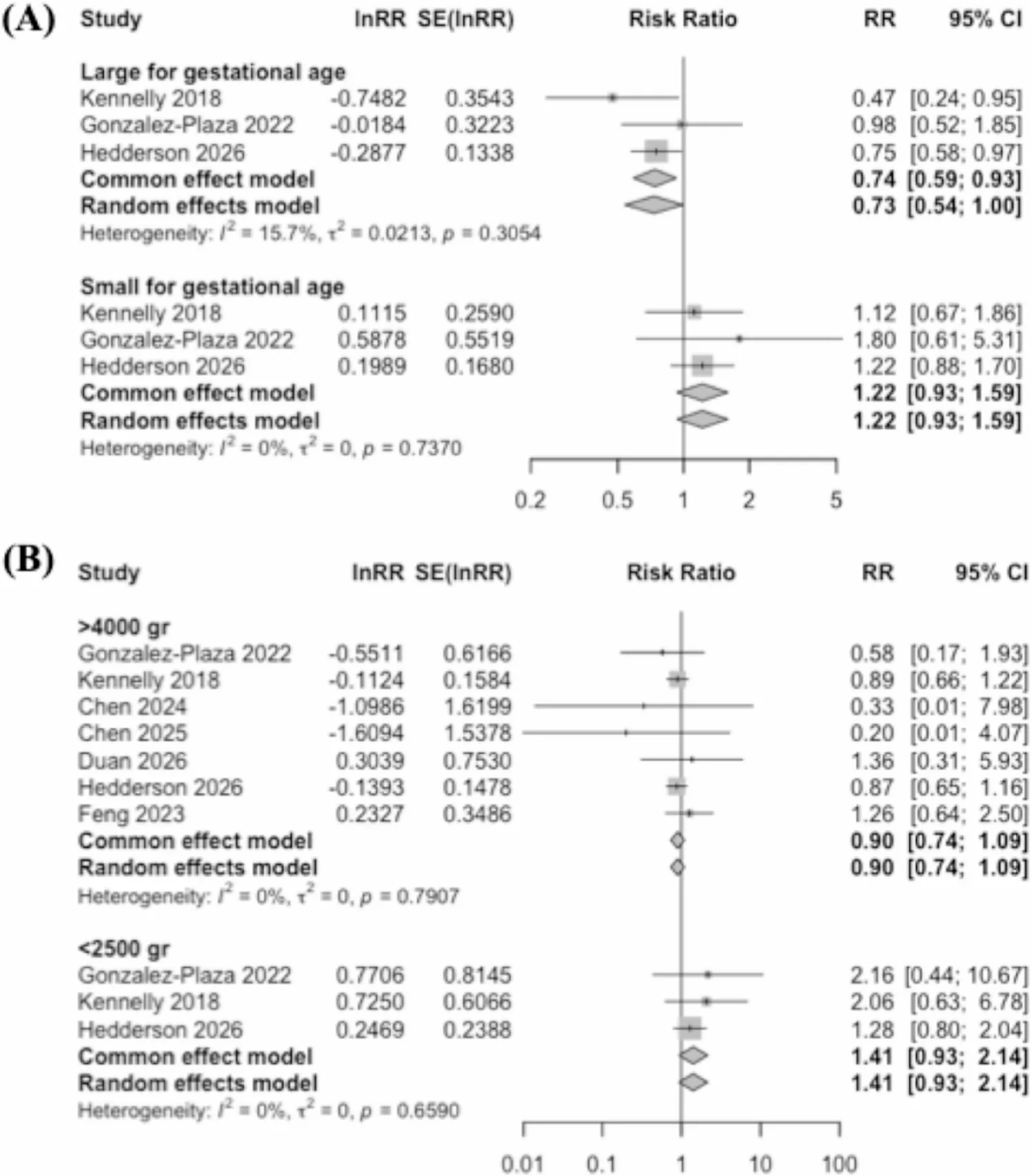
Forest plot of birth weight outcome according to the World Health Organization classification of the newborn **A** Birth weight outcomes based on the 90th and 10th percentile classification of newborns; **B** birth weight outcomes based on the newborn weight thresholds of ≥4,000 g and ≤2,500 g

A similar pattern was observed in analyses using birth-weight cutoffs. Mobile apps probably reduce the risk of birth weight > 4,000 g (RR, 0.90; 95% CI, 0.74–1.09), corresponding to 6 fewer cases per 1,000 newborns (16 fewer to 5 more). In contrast, they may increase the risk of birth weight < 2,500 g (RR, 1.41; 95% CI, 0.93–2.14), corresponding to 8 more cases per 1,000 newborns (1 fewer to 23 more). (Fig. 2).

For birth weight as a continuous outcome, mobile app may result in a mean reduction of 32.44 g (95% CI, 139.64 g lower to 74.75 g higher); however, the estimate was imprecise. (Fig. 4).

### Gestational weight

**Fig. 3:**
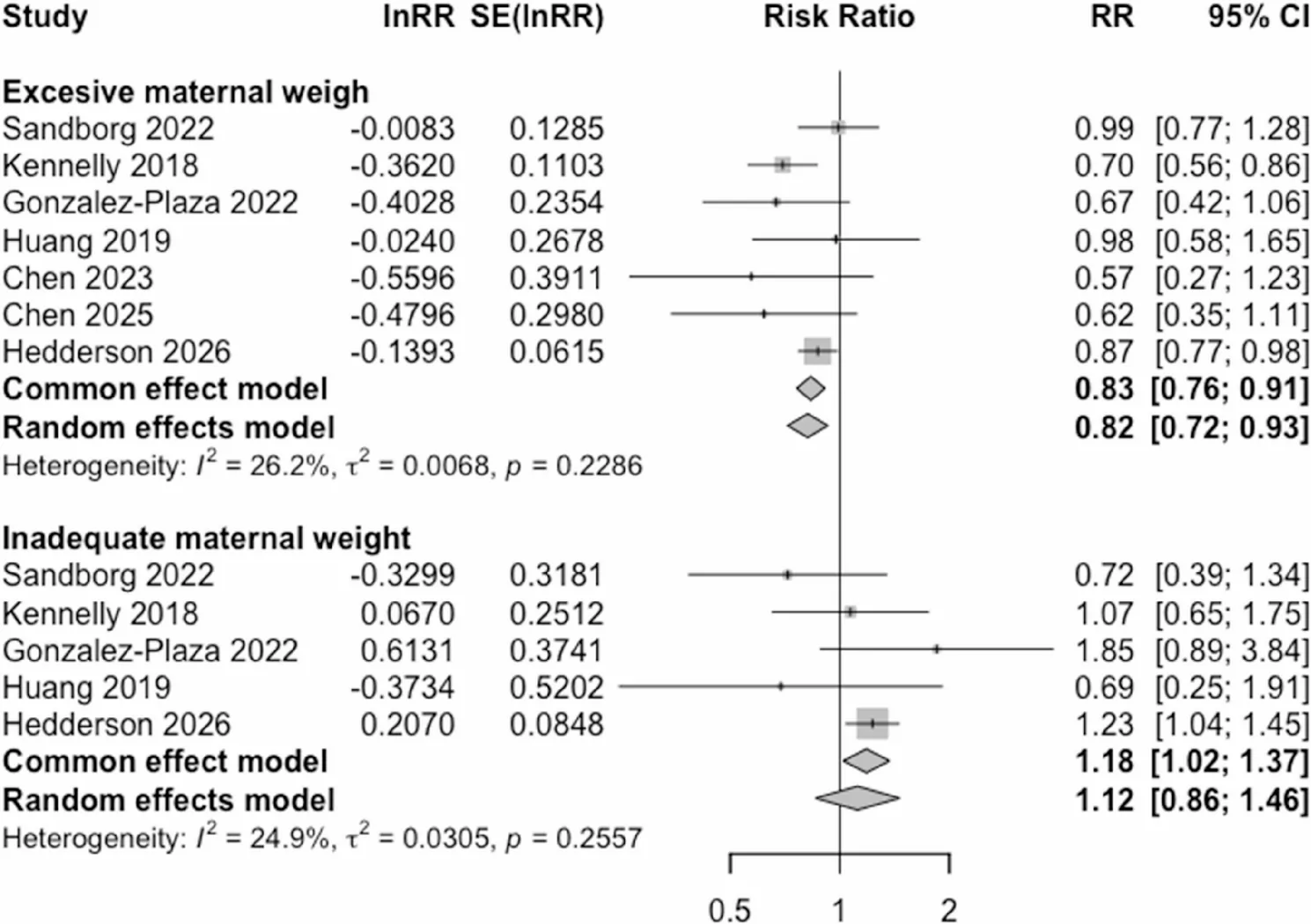
Forest plot of gestational weight outcomes based on the recommendations of the US Institute of Medicine

**Fig. 4:**
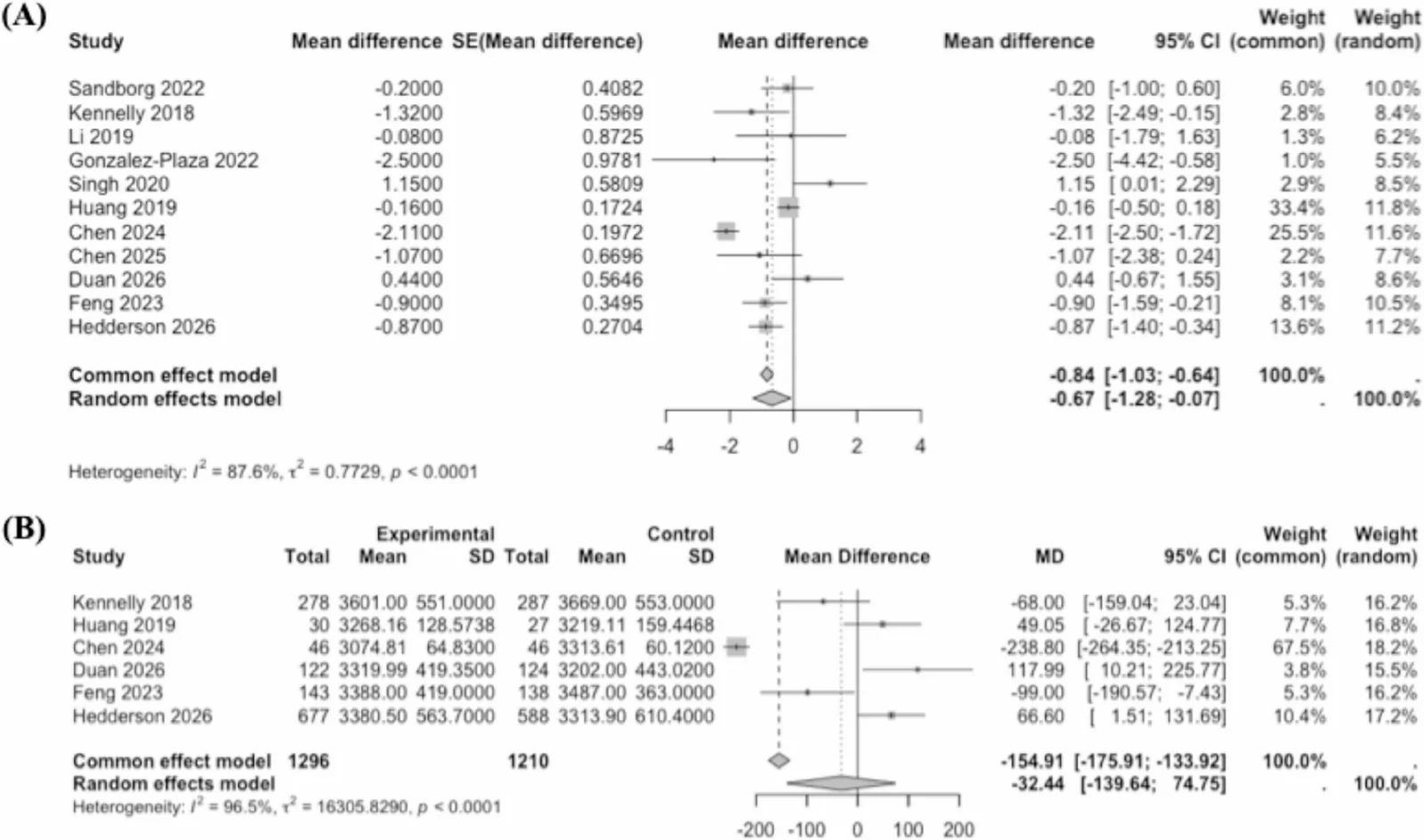
Forest plot of changes in gestational weight gain (**A**) and birth weight (**B**)

Twelve studies assessed gestational weight gain outcomes. Seven reported gestational weight gain as a categorical outcome (excessive or inadequate), whereas 11 reported it as a continuous outcome based on the change in maternal weight. Results showed that mobile apps may reduce the risk of excessive maternal weight (RR 0.82; CI95% 0.72–0.93) but may increase the risk of inadequate (RR 1.12; CI95% 0.86–1.46), resulting in 41 fewer (63 fewer to 16 fewer) and 10 more (11 fewer to 38 more) cases per 1,000, respectively (Fig. 3). When analyzed as a continuous outcome, mobile app may reduce gestational weight gain in 0.67 kg (95% CI, −1.28 to 0.07). (Fig. 4).

### Other maternal anthropometric measurements

**Fig. 5:**
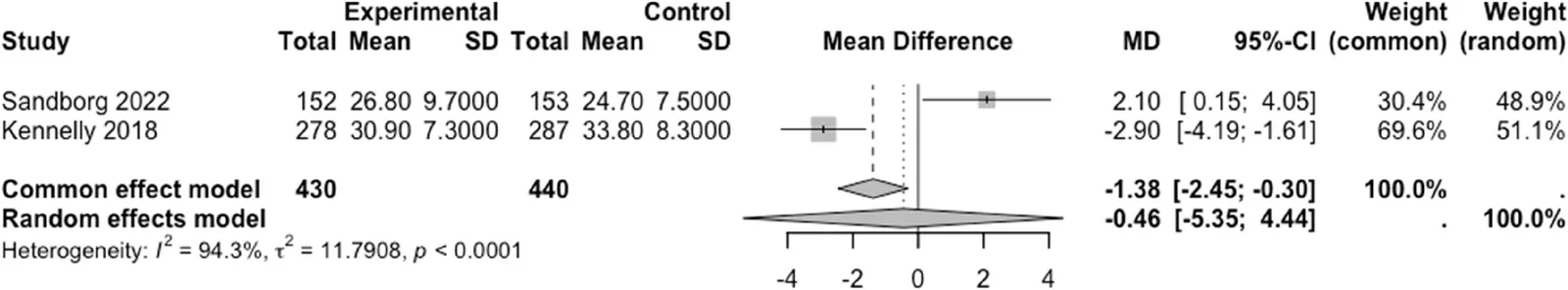
Forest plot of the outcome of body fat percentage (%)

The studies also evaluated other outcomes such as body fat percentage, BMI, and arm circumference. Two studies [31, 32] assessed body fat percentage, however, the results were imprecise. Meta-analysis showed that mobile apps may reduce body fat in 0.46% (MD −0.46; CI95% −5.35 to 4.44) (Fig. 5). One study [11] reported BMI at two follow-up time points. Compared with the control group, the mobile app group showed a greater increase in BMI at both 4 and 8 weeks in 1.60 kg/m² (95% CI, −1.09 to 4.29) and 0.70 kg/m² (95% CI, −1.95 to 3.35), respectively. Two studies [32, 35] also assessed arm circumference, both showing a reduction favoring mobile apps. One study reported change from baseline (MD −0.37 cm; CI95% −0.53 to −0.21), while other only compare values at the end of the follow up (MD −0.2 cm; CI95% −10.44 to 10.04).

### Risk of bias and certainty of the evidence

Most studies contributing to each outcome were judged to be at low risk of bias. One study was rated as having a high risk of bias for the birth-weight and gestational-weight outcomes, mainly because of deviations from the intended interventions and missing outcome data. For birth-weight outcomes, one study raised some concerns because of bias in the selection of the reported result. For gestational-weight outcomes, two studies raised some concerns for the same reason, whereas another study raised some concerns because of deviations from the intended interventions and missing outcome data. (Table 2).

**Table 2:** Risk of bias of included studies by outcome

Outcome	Study	D1	D2	D3	D4	D5	Overall
Birth weight	Kennelly 2018 [32]	Low risk	Low risk	Low risk	Low risk	Low risk	Low risk
	Gonzalez-Plaza 2022 [34]	Low risk	Low risk	Low risk	Low risk	Low risk	Low risk
	Huang 2019 [35]	Low risk	Low risk	Low risk	Low risk	Low risk	Low risk
	Hedderson 2026 [43]	Low risk	Low risk	Low risk	Low risk	Low risk	Low risk
	Chen 2024 [37]	Some concerns	Low risk	High risk	Low risk	Some concerns	High risk
	Chen 2025 [40]	Low risk	Low risk	Low risk	Low risk	Some concerns	Some concerns
	Duan 2026 [41]	Low risk	Low risk	Low risk	Low risk	Low risk	Low risk
	Feng 2023 [42]	Low risk	Low risk	Low risk	Low risk	Low risk	Low risk
Gestational weight	Sandborg 2021 [31]	Low risk	Low risk	Low risk	Low risk	Low risk	Low risk
	Kennelly 2018 [32]	Low risk	Low risk	Low risk	Low risk	Low risk	Low risk
	Li 2019 [11]	Low risk	Low risk	Low risk	Low risk	Low risk	Low risk
	Gonzalez-Plaza 2022 [34]	Low risk	Some concerns	Some concerns	Low risk	Low risk	Some concerns
	Huang 2019 [35]	Low risk	Low risk	Low risk	Low risk	Low risk	Low risk
	Singh 2020 [33]	Low risk	Low risk	Low risk	Low risk	Low risk	Low risk
	Chen 2023 [38]	Some concerns	Low risk	Low risk	Low risk	Some concerns	Some concerns
	Chen 2025 [40]	Low risk	Low risk	Low risk	Low risk	Some concerns	Some concerns
	Hedderson 2026 [43]	Low risk	Low risk	Low risk	Low risk	Low risk	Low risk
	Chen 2024 [37]	Some concerns	Low risk	High risk	Low risk	Some concerns	High risk
	Duan 2026 [41]	Low risk	Low risk	Low risk	Low risk	Low risk	Low risk
	Feng 2023 [42]	Low risk	Low risk	Low risk	Low risk	Low risk	Low risk
Body fat	Sandborg 2021 [31]	Low risk	Low risk	Low risk	Low risk	Low risk	Low risk
	Kennelly 2018 [32]	Low risk	Low risk	Low risk	Low risk	Low risk	Low risk
Body mass index	Li 2019 [11]	Low risk	Low risk	Low risk	Low risk	Low risk	Low risk
Arm circumference	Kennelly 2018 [32]	Low risk	Low risk	Low risk	Low risk	Low risk	Low risk
	Huang 2019 [35]	Low risk	Low risk	Low risk	Low risk	Low risk	Low risk

(D1) Bias due to the random assignment generation process

(D2) Bias due to deviations from planned interventions

(D3) Bias due to missing outcome data

(D4) Bias in outcome measurement

(D5) Bias in the selection of the result report

**Fig. 6:**
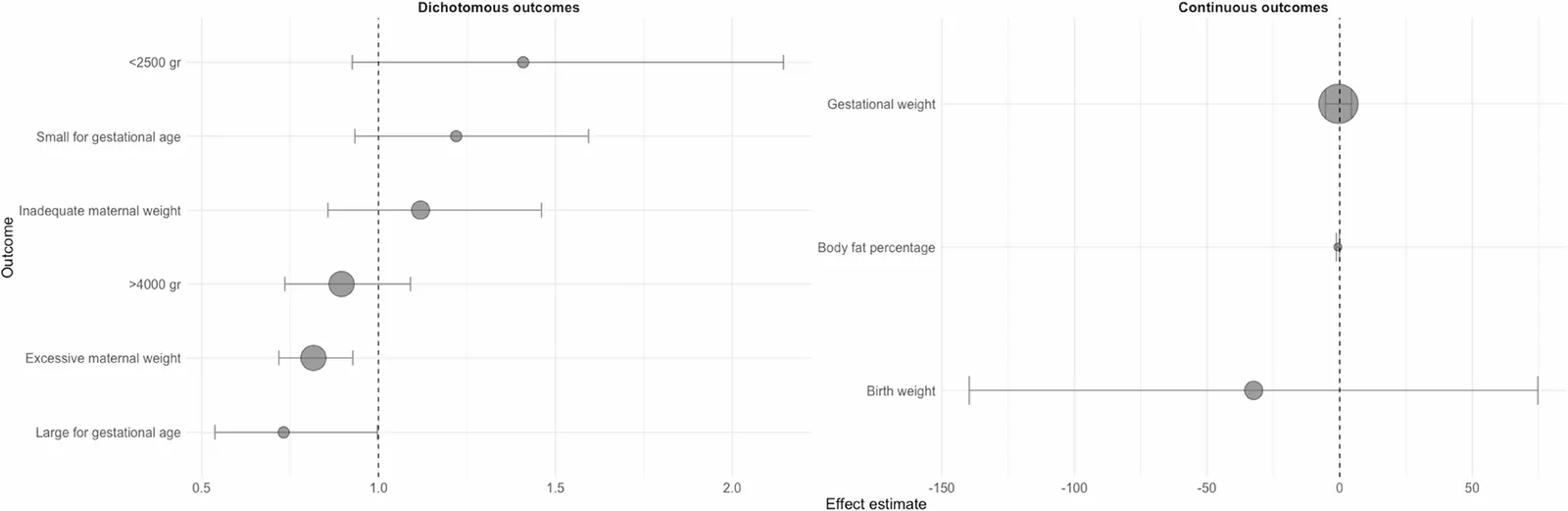
Bubble chart of multiple meta-analysis results

The certainty of the evidence was downgraded primarily because of imprecision, by one to three levels. Across outcomes, certainty ratings ranged from very low to high. Very-low-certainty evidence was identified for birth weight < 2,500 g, body fat percentage, body mass index, and arm circumference. A summary of the results for each outcome is provided in (Table 3) and (Fig. 6).

**Table 3:** Summary Table of Results

Outcomes	Participants Nº studies	Certainty of the evidence (GRADE)**	Relative effect (95% CI)	Anticipated absolute effects		Interpretation***
				Baseline risk	Risk difference with mobile apps	
Birth weight (g)	2506 6 RCTs [32, 35, 37, 41–43]	⨁⨁◯◯ Low ^a, b^	-	-	MD 32.44 g fewer (139.64 fewer to 74.75 more)	Mobile apps may result in little to no difference in birth weight
Birth weight-small for gestational age	1887 3 RCTs [32, 34, 43]	⨁⨁◯◯ Low ^a, b^	RR 1.22 (0.93 to 1.59)	37 per 1000	8 more per 1000 (3 fewer to 22 more)	Mobile apps may result in little to no difference in small for gestational age birth weight.
Birth weight-<2500 g	1940 3 RCTs [32, 34, 43]	⨁◯◯◯ Very low ^a, c^	RR 1.41 (0.93 to 2.14)	20 per 1000	8 more per 1000 (1 fewer to 23 more)	Mobile apps may increase < 2500 birth weight cases slightly, but the evidence is very uncertain.
Birth weight-Large for gestational age	1887 3 RCTs [32, 34, 43]	⨁⨁⨁◯ Moderate ^a, d^	RR 0.73 (0.54 to 1.00)	64 per 1000	17 fewer per 1000 (29 fewer to 0)	Mobile apps probably reduce large for gestational age birth weight cases.
Birth weight->4000 g	2671 7 RCTs [32, 34, 37, 40–43]	⨁⨁⨁◯ Moderate ^a, d^	RR 0.90 (0.74 to 1.09)	60 per 1000	6 fewer per 1000 (16 fewer to 5 more)	Mobile apps probably result in little to no difference in > 4000 g birth weight.
Gestational weight (kg)	3516 11 RCTs [11, 31–35, 37, 40–43]	⨁⨁⨁⨁ High	-	-	MD 0.67 kg fewer (1.28 fewer to 0.07 fewer)	Mobile apps result in little to no difference in gestational weight.
Gestational weight-Excessive maternal weight	2531 7 RCTs [31, 32, 34, 35, 38, 40, 43]	⨁⨁⨁◯ Moderate ^a, d^	RR 0.82 (0.72 to 0.93)	226 per 1000	41 fewer per 1000 (63 fewer to 16 fewer)	Mobile apps probably result in little to no difference in excessive maternal weight.
Gestational weight-Inadequate maternal weight	2327 5 RCTs [31, 32, 34, 35, 43]	⨁⨁⨁◯ Moderate ^a, d^	RR 1.12 (0.86 to 1.46)	82 per 1000	10 more per 1000 (11 fewer to 38 more)	Mobile apps probably result in little to no difference in inadequate maternal weight.
Body fat (%)	870 2 RCTs [31, 32]	⨁◯◯◯ Very low ^c^	-	-	MD 1.41% fewer (4.22 fewer to 1.39 more)	Mobile apps may result in little to no difference in body fat, but the evidence is very uncertain.
Body mass index (kg/m2; 8 weeks of follow up)	30 1 RCT [11]	⨁◯◯◯ Very low ^c^	-	-	MD 0.7 kg/m^2^ more (1.22 fewer to 2.62 more)	Mobile apps may result in little to no difference in body mass index, but the evidence is very uncertain.
Arm circumference	572 2 RCT [32, 35]	⨁◯◯◯ Very low ^c^	Two studies assessed arm the outcome, but in both cases favored mobile apps. One study reported a greater reduction from baseline in 0.37 cm (95% CI, −0.53 to −0.21), whereas the other reported a greater reduction at the end of follow-up in −0.20 cm (95% CI, −10.44 to 10.04).			Mobile apps result in little to no difference in arm circumference.

*CI* Confidence interval, *MD* Mean difference, *RR* Risk ratio

^a^Most of the studies were considered low risk of bias, even there were some with some concerns or high risk the weight into the estimation is low

^b^Downgrade two levels due to serious imprecision, considering the estimation crosses two thresholds

^c^Downgrade three levels due to very serious imprecision, considering the estimation crosses three thresholds

^d^Downgrade one level due to imprecision, considering the estimation crosses one threshold

*The risk in the intervention group (and its 95% confidence interval) is based on the assumed risk in the comparison group (baseline risk) and the relative effect of the intervention (and its 95% CI)

**Thresholds were defined in increments of the minimally important difference (MID); therefore, each additional change equivalent to one MID constituted a new threshold. For dichotomous outcomes, a 25% relative change was considered clinically important, and the corresponding absolute thresholds were calculated using the baseline risk for each outcome

***Interpretations considered both the certainty of the evidence and the magnitude of effect according to the prespecified thresholds

## Discussion

Although the use of mobile apps during pregnancy has generated interest as a potential tool to promote healthy lifestyles, the clinical trial results on their efficacy for controlling gestational weight are inconclusive.

While these tools are expected to be more flexible and promote better adherence, our study found that adherence to mobile app use varied across included studies, reaching up to 60%. This is particularly relevant, as adherence plays a key role in the efficacy of lifestyle-change interventions [44]. Although some studies reported daily use by participants, they did not specify the duration or frequency of use. Others failed to provide usage details altogether or reported intermittent or unquantifiable use. This variability in app engagement measurement limits the comparability of real effects and may contribute to the heterogeneity of the results. Nevertheless, we believe that adherence to the intervention was likely higher, particularly because it was assessed within the context of a clinical trial, and higher than that observed in routine prenatal care, as supported by evidence showing that adherence rates rarely exceed 50% [11, 33, 34]. To implement mobile apps, use in real-world settings, additional strategies are necessary to improve and maintain sustained engagement, particularly because studies have shown a progressive decline in user commitment over time [31, 32, 35]. Such strategies may include remote professional support [34], automated and personalized feedback [31, 36], digital social support [36], and simple reminders via text messages [33].

Our study identified a paradoxical situation in which mobile apps reduced the risk of excessive maternal and neonatal weight but increased the risk of weights below the recommended threshold. This outcome may be attributed to the characteristics of the evaluated interventions, which frequently provided generalized recommendations that were not tailored to individual needs. Most interventions lacked clinical supervision and failed to incorporate adjustments based on BMI, nutritional status, or specific medical conditions that emerged during pregnancy. As a result, these factors may have contributed to excessive or inadequate caloric restriction, particularly among women without overweight. Additionally, some apps lacked specific guidance on macronutrient requirements, possibly resulting in nutritional imbalances and affecting maternal weight and fetal development. These findings reinforce the need for digital interventions to be complemented by professional medical and nutritional oversight, as the complexity of developing truly individualized recommendations within mobile apps makes it unlikely that these tools alone can ensure safe and effective outcomes. Indeed, some studies have evaluated hybrid approaches integrating mobile technologies with clinical care, showing that such models may be more effective and safer than app-only interventions [45].

Regarding other anthropometric outcomes, most showed a trend toward the expected reduction in measurements, except for BMI outcome. This result may be explained by several factors. The study that assessed this outcome was a pilot trial with a small sample size, limiting statistical power and increasing susceptibility to individual variability [31]. Also, the intervention has a short follow-up (8 weeks) and started in the second half of pregnancy, a stage when BMI tends to increase physiologically. Moreover, the app used required participants to upload photos of their meals to receive feedback. The effectiveness of this intervention depended heavily on participants’ honesty and consistency in meal reporting, as well as the clarity and applicability of the feedback. These limitations may have compromised the intervention’s ability to induce meaningful dietary changes and, consequently, influence BMI outcome.

It is important to note that, in most studies, the intervention was not limited to a mobile app but also included additional components, such as coaching and educational sessions, that were not part of standard care. Consequently, it remains uncertain whether the observed effects can be attributed specifically to the mobile apps or to the combined intervention strategies. Nevertheless, evidence from reviews of mobile app-based interventions in other populations has shown consistent findings, lending plausibility to the hypothesis that mobile apps contributed to the effects observed in our review. For example, a review conducted in the general population [46] concluded that mobile apps may support the adoption of healthy behaviors, although their effects on anthropometric outcomes varied across outcomes. Similarly, a systematic review of pregnant women with gestational diabetes [47] reported improvements in treatment adherence and glycemic control associated with mobile app use.

### Implications for decision-making

Although a reduction in the risk of excessive maternal weight and potential benefits in other neonatal and anthropometric measures has been observed, these results do not translate into clinically significant effects. Therefore, the use of mobile apps could represent a promising tool for managing gestational weight, but only in populations with limited access to in-person healthcare services, such as pregnant women with disabilities. However, the potential increase in the risk of inadequate maternal weight and low birth weight highlights the need for careful monitoring of these interventions to avoid unintended adverse effects. For effective implementation, these strategies should incorporate mechanisms that support sustained adherence over time, as well as ensure equitable access to technology, with particular attention to the protection of personal data [31]. Although we have not identified technical documents or clinical practice guidelines evaluating the use of mobile apps for monitoring pregnant populations, these findings could provide evidence to inform decision-making.

### Limitations

This study has several limitations. A major limitation is the methodological heterogeneity among interventions and how mobile apps were implemented, including frequency of use, type of educational content, and monitoring tools, which may have influenced the magnitude of observed effects. Furthermore, adherence to the intervention is a potential source of bias. The efficacy of mobile apps largely depends on user commitment, and the lack of strict control over actual usage may have influenced the results. Some studies did not objectively measure the frequency and quality of app use, making it difficult to assess their true impact on outcomes.

## Conclusions

Mobile apps may represent a promising adjunct for weight management during pregnancy; however, the available evidence remains heterogeneous. Although these interventions may modestly reduce gestational weight gain and neonatal birth weight, they may also increase the likelihood of maternal and neonatal weight outcomes falling below recommended ranges. Moreover, the observed effects were not clinically meaningful for most outcomes. It is pertinent to explore the use of these interventions in combination with professional follow-up strategies, using a multifaceted approach that integrates behavioral, social, and environmental dimensions related to weight management and that favors personalization of the user experience. Additionally, user-centered design that incorporates interactive elements and adaptive features may improve adherence, engagement, and efficacy of these digital tools, thereby optimizing outcomes related to gestational weight control.

## Supplementary Information

Supplementary Material 1.

## Data Availability

No datasets were generated or analysed during the current study.

## Abbreviations

BMI: Body mass index
CI: Confidence interval
GRADE: Grading of recommendations assessment, development and evaluation
GWG: Gestational weight gain
LBW: Low birth weight
MeSH: Medical subject headings
PRISMA: Preferred reporting items for systematic reviews and meta-analyses
RCT: Randomized controlled trial
RoB 2: Risk of bias 2 tool
RR: Risk ratio
SD: Standard deviation
