# Supplementary material for: Effects of mobile apps on maternal weight-related and neonatal birth weight outcomes during pregnancy: a systematic review and meta-analysis
Source: BMC Pregnancy Childbirth. 2026 Aug 13;26:1304. doi: 10.1186/s12884-026-09778-8 (PMC13749800; doi:10.1186/s12884-026-09778-8)
Supplement: Supplementary file 1 [file 12884_2026_9778_MOESM1_ESM.docx]

# SUPPLEMENTARY MATERIAL

## Supplementary 1. PRISMA 2020 Checklist

| **Section/Topic** | | | **#** | **Item** | | **Present on page #** |
| --- | --- | --- | --- | --- | --- | --- |
| **TITLE** | | | | | |  |
| Title | | | 1 | Identify the publication as a systematic review, meta-analysis, or both. | | 1 |
| **SUMMARY** | | | | | |  |
| Structured Summary | | | 2 | Provide a structured abstract that includes, as appropriate: background; Objectives; source of the data; eligibility criteria for studies, participants and interventions; evaluation of studies and synthesis methods; results; Limitations; conclusions and implications of the main findings; Systematic review registration number. | | 2 |
| **INTRODUCTION** | | | | | |  |
| Justification | | | 3 | Describe the rationale for the review in the context of what is already known about the topic. | | 3 |
| Objectives | | | 4 | Explicitly state the questions to be answered in relation to participants, interventions, comparisons, results and study design (PICOS). | | 4 |
| **METHODS** | | | | | |  |
| Protocol and registration | | | 5 | Indicate whether there is a review protocol that can be accessed (e.g., web address) and, if available, information about the record, including its registration number. | | 4 |
| Eligibility Criteria | | | 6 | Specify the characteristics of the studies (e.g. PICOS, duration of follow-up) and characteristics (e.g. years covered, languages or publication status) used as eligibility criteria and their justification. | | 4 and 5 |
| Sources of information | | | 7 | Describe all sources of information (e.g. databases and search periods, contact with authors to identify additional studies, etc.) in the search and the date of the last search conducted. | | 4 |
| Search | | | 8 | Present the complete electronic search strategy in at least one database, including the limits used in such a way that it can be reproducible. | | 4 |
| Study selection | | | 9 | Specify the selection process for studies (e.g. screening and eligibility included in the systematic review and, where relevant, included in the meta-analysis). | | 5 |
| Data collection process | | | 10 | Describe methods for extracting data from publications (e.g., directed, duplicate, and independent forms) and any process for obtaining and confirming data by researchers. | | 5 |
| Data List | | | 11 | List and define all variables for which data were searched (e.g. PICOS funding source) and any assumptions and simplifications that have been made. | | 4 – 5 |
| Risk of bias in individual studies | | | 12 | Describe the methods used to assess risk of bias in individual studies (specify whether it was conducted at the study or outcome level) and how this information has been used in data synthesis. | | 5 |
| Summary measures | | | 13 | Specify the main summary measures (e.g. risk ratio or mean difference). | | 6 and 7 |
| Summary of results | | | 14 | Describe methods for handling data and combining study results, if any, including measures of consistency (e.g., I2) for each meta-analysis. | | 6 and 7 |
| Risk of bias between studies | | | 15 | Specify any assessments of the risk of bias that may affect cumulative evidence (e.g. publication bias or selective communication). | | 5 |
| Additional Analysis | | | 16 | Describe additional methods of analysis (e.g., sensitivity or subgroup analysis, meta-regression), if any, indicate which ones were pre-specified. | | 7 |
|  | **RESULTS** | | | | |  |
|  | Study selection | 17 | | | Provide the number of studies screened, assessed for eligibility and included in the review, and detail the reasons for their exclusion at each stage, ideally using a flowchart. | 7 |
|  | Study characteristics | 18 | | | For each study, present the characteristics for which data were extracted (e.g. size, peaks, and duration of follow-up) and provide bibliographic citations. | 7 |
|  | Risk of bias in studies | 19 | | | Present data on risk of bias in each study and, if available, any assessment of bias in the results (see item 12). | 9, 10, and 26 |
|  | Results of individual studies | 20 | | | For each outcome considered for each study (benefits or harms), present: a) the summary data for each intervention group and b) the estimate of the effect with its confidence interval, ideally graphically using a forest plot. | 9, 24 and 25 |
|  | Summary of the results | 21 | | | To present results of all meta-analyses performed, including confidence intervals and measures of consistency. | 8,9, 24 and 25 |
|  | Risk of bias between studies | 22 | | | Present the results of any assessment of risk of bias between studies (see item 15). | 9 and 10 |
|  | Additional Analysis | 23 | | | Provide the results of any additional analyses, if they have been performed (e.g., sensitivity or subgroup analyses, meta-regression [see item 16]) | - |
|  | **DISCUSSION** | | | | |  |
|  | Summary of the evidence | 24 | | | Summary the main findings, including the strength of the evidence for each main outcome; consider their relevance to key groups (e.g., caregivers, users, and health decision-makers). | 8-13 |
|  | Limitations | 25 | | | To discuss the limitations of the studies and outcomes (e.g. risk of bias) and of the review (e.g. incomplete sourcing of identified studies or selective reporting). | 13 |
|  | Conclusions | 26 | | | Provide a general interpretation of the results in the context of other evidence, as well as implications for future research. | 13 |
|  | **FINANCING** | | | | |  |
|  | Financing | 27 | | | Describe the sources of funding for the systematic review and other support (e.g. data input), as well as the role of funders in the systematic review. | 14 |

## Supplementary 2. Search strategy

| **Database** | **Search strategy** | **Number of studies** |
| --- | --- | --- |
| PUBMED | #1="Pregnant People"[Mesh] OR "Pregnancy"[Mesh] OR pregnant*[tiab] OR gestation*[tiab] OR gravidity[tiab]  #2= "Mobile Applications"[Mesh] OR "Telemedicine"[Mesh] OR ((mobile[tiab] OR smartphone[tiab] OR cellphone[tiab]) AND (app*[tiab] OR application*[tiab] OR health[tiab])) OR e-Health[tiab] OR eHealth[tiab] OR "digital healthcare app*"[tiab] OR mHealth[tiab] OR "portable software app*"[tiab] OR "tablet app*"[tiab] OR telehealth[tiab] OR "tele-health"[tiab] OR telemedicine[tiab]  #3="Obesity"[Mesh] OR "Pregnancy in Obesity"[Mesh] OR "Overweight"[Mesh] OR "Malnutrition"[Mesh] OR "Diet"[Mesh] OR "Body Weight"[Mesh] OR "Body Weight Changes"[Mesh] OR "Exercise"[Mesh] OR "maternal obesity"[tiab] OR "obesity in pregnancy"[tiab] OR obesity[tiab] OR overweight[tiab] OR "body weight*"[tiab] OR "body weight change"[tiab] OR undernutrition[tiab] OR malnourishment*[tiab] OR malnutrition[tiab] OR "healthy weight gain"[tiab] OR "gestational weight gain"[tiab] OR diet*[tiab] OR "optimal weight"[tiab] OR "weight loss"[tiab] OR "weight gain prevention"[tiab] OR exercise*[tiab] OR ((physical[tiab] OR Aerobic[tiab] OR isometric[tiab] OR fitness[tiab]) AND (activit*[tiab] OR exercise*[tiab] OR training[tiab] OR workout[tiab])) | 416  (Update: 315) |
| SCOPUS | #1= TITLE-ABS-KEY (pregnant* OR gestation* gravidity)  #2= TITLE-ABS-KEY(Telemedicine OR ((mobile OR smartphone OR cellphone) AND (app* OR application* OR health)) OR e-Health OR eHealth OR "digital healthcare app*" OR mHealth OR "portable software app*" OR "tablet app*" OR telehealth OR "tele-health")  #3= TITLE-ABS-KEY(Obesity OR Overweight OR Malnutrition OR Diet* OR "Body Weight*" OR "Body Weight Changes" OR Exercise* OR "maternal obesity" OR "obesity in pregnancy" OR undernutrition OR malnourishment* OR "healthy weight gain" OR "gestational weight gain" OR "optimal weight" OR "weight loss" OR "weight gain prevention" OR ((physical OR Aerobic OR isometric OR fitness) AND (activit* OR exercise* OR training OR workout))) | 2  (Update: 0) |
| WEB OF SCIENCE | #1= TS=(pregnant* OR gestation* gravidity)  #2= TS=(Telemedicine OR ((mobile OR smartphone OR cellphone) AND (app* OR application* OR health)) OR e-Health OR eHealth OR "digital healthcare app*" OR mHealth OR "portable software app*" OR "tablet app*" OR telehealth OR "tele-health")  #3= TS=(Obesity OR Overweight OR Malnutrition OR Diet* OR "Body Weight*" OR "Body Weight Changes" OR Exercise* OR "maternal obesity" OR "obesity in pregnancy" OR undernutrition OR malnourishment* OR "healthy weight gain" OR "gestational weight gain" OR "optimal weight" OR "weight loss" OR "weight gain prevention" OR ((physical OR Aerobic OR isometric OR fitness) AND (activit* OR exercise* OR training OR workout))) | 363  (Update: 205) |
| EMBASE | #1 ('pregnancy'/exp/mj OR 'child bearing':ti,ab OR 'childbearing':ti,ab OR 'gestation':ti,ab OR 'gravidity':ti,ab OR 'intrauterine pregnancy':ti,ab OR 'labor presentation':ti,ab OR 'labour presentation':ti,ab OR 'pregnancy':ti,ab OR 'pregnancy maintenance':ti,ab OR 'pregnancy trimesters':ti,ab OR 'pregnant woman'/exp/mj OR 'pregnant woman': ti,ab OR 'pregnant women':ti,ab)  #2 ('mobile application'/exp/mj OR 'mobile app':ti,ab OR 'mobile application':ti,ab OR 'mobile applications':ti,ab OR 'mobile apps':ti,ab OR 'portable software app':ti,ab OR 'portable software application':ti,ab OR 'portable software applications':ti,ab OR 'portable software apps':ti,ab OR 'tablet application':ti,ab OR 'telehealth'/mj OR 'e-health': ti,ab OR 'ehealth':ti,ab OR 'tele-health':ti,ab OR 'telehealth':ti,ab OR ('mobile phone'/exp/mj OR 'cell phone':ti,ab OR 'cell phones':ti,ab OR 'cellphone':ti,ab OR 'cellphones':ti,ab OR 'cellular phone':ti,ab OR 'cellular telephone':ti,ab OR 'mobile phone':ti,ab OR 'mobile telephone':ti, ab OR 'smartphone'/exp/mj OR 'smart phone':ti,ab OR 'smartphone':ti,ab OR 'smartphones':ti,ab) AND (app:ti,ab OR 'application'/exp/mj OR 'health'/mj)))  #3 ('obesity'/exp/mj OR 'adipose tissue hyperplasia':ti,ab OR 'adipositas':ti,ab OR 'adiposity':ti,ab OR 'alimentary obesity':ti,ab OR 'body weight, excess':ti,ab OR 'corpulency':ti,ab OR 'fat overload syndrome':ti,ab OR 'nutritional obesity':ti,ab OR 'obesitas':ti,ab OR 'obesity':ti,ab OR 'overweight':ti,ab OR 'maternal obesity'/exp/mj 'maternal obesity': ti,ab OR 'obesity, maternal':ti,ab OR 'malnutrition'/exp/mj OR 'deficient nutrition':ti,ab OR 'malnourishment':ti,ab OR 'malnutrition':ti,ab OR 'severe acute malnutrition':ti,ab OR 'underfeeding':ti,ab OR 'undernourishment':ti,ab OR 'undernutrition':ti,ab OR 'diet'/mj OR 'diet':ti,ab OR 'diet influence':ti,ab OR 'diet regimen': ti,ab OR 'diet surveys':ti,ab OR 'dietary effect':ti,ab OR 'dietary influence':ti,ab OR 'dietary survey':ti,ab OR 'dietary surveys':ti,ab OR 'dieting':ti,ab OR 'body weight change'/exp/mj OR 'body weight change':ti,ab OR 'body weight changes':ti,ab OR 'weight change':ti,ab OR 'weight changes': ti,ab OR 'exercise'/mj OR 'biometric exercise':ti,ab OR 'effort':ti,ab OR 'exercise':ti,ab OR 'exercise capacity':ti,ab OR 'exercise performance':ti,ab OR 'exercise training':ti,ab OR 'exertion':ti,ab OR 'fitness training':ti,ab OR 'fitness workout':ti,ab OR 'physical conditioning, human':ti,ab OR 'physical effort': ti,ab OR 'physical exercise':ti,ab OR 'physical exertion':ti,ab OR 'physical work-out':ti,ab OR 'physical workout':ti,ab OR 'obesity in pregnancy'/exp/mj OR 'body weight'/exp/mj OR 'body weight':ti,ab OR 'total body weight':ti,ab OR 'weight, body':ti,ab OR 'healthy weight gain':ti,ab OR 'gestational weight gain'/exp/mj OR 'gestational weight gain': ti,ab OR 'pregnancy weight gain':ti,ab OR 'optimal weight':ti,ab OR 'body weight loss'/exp/mj OR 'body weight decrease' OR 'body weight loss' OR 'body weight reduction' OR 'weight decrease' OR 'weight losing' OR 'weight loss' OR 'weight reducing' OR 'weight reduction' OR 'weight watching' OR 'weight gain prevention':ti,ab) | 181  (Update: 400) |

## Supplementary 3. Excluded studies

| **ID** | **Studies** | **Reason for exclusion** |
| --- | --- | --- |
| Abbaspoor 2020 | The effect of education through mobile phone short message service on promoting self-care in pre-diabetic pregnant women: A randomized controlled trial | Wrong population. 100% population with metabolic disease |
| Ainscough 2016 | Impact of an mHealth supported healthy lifestyle intervention on behavioral stage of change in overweight and obese pregnancy | Wrong study design |
| Ainscough 2017 | Impact of an mHealth-supported behavioral lifestyle intervention on exercise stage-of-change and physical activity in overweight and obese pregnancy: PEARs randomized controlled trial (RCT) | Not available in its full version |
| Angeby 2025 | Testing the ELSA Birth App During Pregnancy and Labor for Primiparous Women: Randomized Controlled Trial | Wrong outcome |
| Asadollahi 2026 | The impact of mHealth interventions on health-promoting behaviors in pregnant women: a randomized clinical trial | Wrong publication type. Article in press |
| Bernardo 2024 | Feasibility, Clinical Efficacy, and Maternal Outcomes of a Remote Exercise Program in Pregnant Women with Obesity: The GROB Randomized Control Pilot Study | Wrong outcome |
| Blackwell 2020 | Using Text Messaging to Improve Access to Prenatal Health Information in Urban African American and Afro-Caribbean Immigrant Pregnant Women: Mixed Methods Analysis of Text4baby Usage | Wrong study design |
| Boehlke 2026 | A cluster-randomised controlled trial evaluating the effects of a blended care intervention in pregnancy and postpartum on weight, health behaviour, and mental health in women with low socio-economic status | Wrong outcome. Self-reporting outcome |
| Bogaerts 2020 | Development and Field Evaluation of the INTER-ACT App, a Pregnancy and Interpregnancy Coaching App to Reduce Maternal Overweight and Obesity: Mixed Methods Design | Wrong study design |
| Bogaerts 2025 | Interpregnancy and pregnancy lifestyle intervention (INTER-ACT): a randomized controlled trial | Wrong population |
| Borgen 2019 | Effect of the Pregnant+ smartphone application in women with gestational diabetes mellitus: A randomized controlled trial in Norway | Wrong population. 100% population with metabolic disease |
| Caballero-Ruiz 2017 | A web-based clinical decision support system for gestational diabetes: Automatic diet prescription and detection of insulin needs | Wrong population. 100% population with metabolic disease |
| Cabre 2025 | Examining the Impact of a Codeveloped Multicomponent Mobile eHealth Lifestyle Intervention on Physical Activity and Its Association With Gestational Weight Gain in Underserved Women: A Statewide Randomized Controlled Trial | Wrong study design. Secondary analysis of RCT |
| Choi 2016 | mHealth Physical Activity Intervention: A Randomized Pilot Study in Physically Inactive Pregnant Women | Wrong outcome. No weight monitoring |
| Deruelle 2020 | Rationale and design of ePPOP-ID: A multicenter randomized controlled trial using an electronic-personalized program for obesity in pregnancy to improve delivery | Wrong outcome. Assess postpartum weight |
| Dieteren 2022 | Impact evaluation of a smartphone application to improve maternal health knowledge and diet among pregnant women in India | Wrong study design |
| Ding 2021 | Evaluation of a Novel WeChat Applet for Image-Based Dietary Assessment among Pregnant Women in China | Wrong outcome. No weight monitoring |
| Dodd 2018 | Evaluation of a smartphone nutrition and physical activity application to provide lifestyle advice to pregnant women: The SNAPP randomized trial | Wrong outcome. No weight monitoring |
| Downs 2021 | Adaptive, behavioral intervention impact on weight gain, physical activity, energy intake, and motivational determinants: results of a feasibility trial in pregnant women with overweight/obesity | Wrong study design |
| Falkenhain 2025 | Effect of a Perinatal eHealth Weight Intervention by Race: Secondary Analysis of 'SmartMoms in WIC' | Wrong publication type |
| Ferrara 2023 | The Effects of a Co-developed Perinatal e-health Intervention for Underserved Women on Maternal Diet: A Secondary Analysis of the “SmartMoms in WIC” Randomized Clinical Trial | Wrong publication type |
| Flanagan 2025 | Correction to Lancet Diabetes Endocrinol 2020; 8: 490–500 | Wrong publication type |
| Flanagan 2025 | Gestational Weight Gain Management in Underserved Mothers: A State-Wide Randomized Trial in WIC | Wrong publication type |
| Gangachin 2024 | Smartphone‑based educational and counseling interventions for women with high body mass index at Urmia’s health centers | Wrong population. No pregnant women |
| Ganju 2016 | Preventing excessive gestational weight gain by using connected weight scales paired with mobile apps | Not available in its full version |
| Garnweidner-Holme 2015 | Designing and Developing a Mobile Smartphone Application for Women with Gestational Diabetes Mellitus Followed Up at Diabetes Outpatient Clinics in Norway | Wrong study design |
| Garnweidner-Holme 2020 | Effect of the Pregnant+ Smartphone App on the Dietary Behavior of Women with Gestational Diabetes Mellitus: Secondary Analysis of a Randomized Controlled Trial | Wrong population. 100% population with metabolic disease |
| Gorjian 2026 | Telephone-based lifestyle education to prevent gestational diabetes in high-risk Iranian women: a randomized trial | Wrong intervention |
| Greene 2018 | Do pregnant women with overweight and obesity find a nutrition and exercise intervention with smartphone app support acceptable? Findings from the PEARs randomized control trial | Wrong outcome. No weight monitoring |
| Greene 2021 | Acceptability of the Pregnancy, Exercise, and Nutrition Research Study with Smartphone App Support (PEARS) and the Use of Mobile Health in a Mixed Lifestyle Intervention by Pregnant Obese and Overweight Women: Secondary Analysis of a Randomized Controlled Trial | It only measures acceptability of the intervention group of the use of the app |
| Guo 2019 | Evaluating the effects of mobile health intervention on weight management, glycemic control and pregnancy outcomes in patients with gestational diabetes mellitus | Wrong population. 100% population with metabolic disease |
| Hassdenteufel 2025 | Improving Maternal Mental Health and Weight Control With a Mindfulness Blended Care Approach: Insights From a Randomized Controlled Trial | Wrong publication type. Article in press |
| His 2021 | Effectiveness of a Nurse-Led Web-Based Health Management in Preventing Women with Gestational Diabetes from Developing Metabolic Syndrome | Wrong population. 100% population with metabolic disease |
| Hojeij 2022 | Effectiveness of Ehealth Coaching Using Smarter Pregnancy on Improving Dietary and Other Lifestyle-Related Behaviors: Can Attitudes Predict Practices? | Not available in its full version |
| Horwitz 2023 | The STAR-MAMA RCT: Bilingual Mobile Health Coaching for Postpartum Weight Loss | Wrong population |
| Huang 2026 | Effects of Peer-Led Walking With mHealth Technology on Exercise Behavior of Women With Overweight and Obesity From Pregnancy to Early Postpartum: A Randomized Controlled Trial | Wrong outcome. No weight monitoring |
| Huberty 2017 | Dose and timing of text messages for increasing physical activity among pregnant women: a randomized controlled trial | Wrong outcome. No weight monitoring |
| Jaeger 2025 | Adherence to digital pregnancy care – lessons learned from the SMART start feasibility study | Wrong outcome |
| Kebbe 2025 | An eHealth Intervention in Pregnancy on Maternal Body Composition and Subsequent Perinatal Outcomes: A Randomized Trial | Wrong intervention. Not specific app-based intervention |
| Kiani 2021 | Mobile-application intervention on physical activity of pregnant women in Iran during the COVID-19 epidemic in 2020 | Wrong study design |
| Killeen 2022 | The Edmonton Obesity Staging System and pregnancy outcomes in women with overweight or obesity: A secondary analysis of a randomized controlled trial | No weight monitoring |
| Knight-Agarwal 2015 | Development and Pilot Testing of the Eating4two Mobile Phone App to Monitor Gestational Weight Gain | Wrong study design |
| Koeryaman 2023 | The Effectiveness of a Web-Based Application for a Balanced Diet and Healthy Weight Among Indonesian Pregnant Women: Randomized Controlled Trial | Wrong intervention |
| Koivuniemi 2023 | Characterising the use, users and effects of a health app supporting lifestyle changes in pregnant women | Wrong comparator |
| Kracht 2026 | Effect of a Pragmatic eHealth Behavioral Gestational Weight Gain Intervention on Household Chaos in Pregnant People of Lower Socioeconomic Status: Randomized Controlled Trial | Wrong outcome. No weight monitoring |
| LaflÈChe 2025 | Associations Between the SmartMoms Canada Mobile Application Usage and Adherence to Gestational Weight Gain Recommendations, Lifestyle Habit Changes and Patient-Centred Outcomes in Pregnant Individuals | Wrong publication type |
| Larsen 2020 | Feasibility and Acceptability of a Counseling- and mHealth-Based Physical Activity Intervention for Pregnant Women with Diabetes: The Fit for Two Pilot Study | Wrong population. 100% population with metabolic disease |
| Leonard 2023 | Feasibility and user acceptability of BreezingTM, a mobile indirect calorimetry device, in pregnant women with overweight or obesity | Wrong outcome |
| Lewkowitz 2014 | Social media messaging in pregnancy: How Text4Baby compares to popular smart phone applications | Wrong study design |
| Li 2025 | [A randomized controlled trial of weight management based on mobile health techno-logy among overweight or obese pregnant women] | Not available in its full version |
| Lim 2021 | A Smartphone App to Restore Optimal Weight (SPAROW) in Women with Recent Gestational Diabetes Mellitus: Randomized Controlled Trial | Wrong population. 100% population with metabolic disease |
| Litman 2022 | Remote gestational weight gain monitoring in a large low-risk US population | Wrong study design |
| Lynch 2024 | Diet quality and blood pressure among pregnant women with overweight or obesity: A secondary analysis of two randomized controlled trials | Wrong study design |
| Mackillop 2018 | Comparing the Efficacy of a Mobile Phone-Based Blood Glucose Management System with Standard Clinic Care in Women with Gestational Diabetes: Randomized Controlled Trial | Wrong population. 100% population with metabolic disease |
| Marko 2016 | Evaluating the Feasibility of Remote Patient Monitoring in Prenatal Care Using a Mobile App and Connected Devices: A Prospective Observational Trial | Wrong study design |
| Martin 2025 | Nutritionist-Guided Video Intervention Improves Adherence to Mediterranean Diet and Reduces the Rate of Gestational Diabetes Mellitus: A Randomized Clinical Trial | Wrong intervention |
| Miller 2024 | Weight Gain Prevention Outcomes From a Pragmatic Digital Health Intervention With Community Health Center Patients: Randomized Controlled Trial | Wrong population |
| Mo 2022 | The Effect of Nursing Intervention Model Using Mobile Nursing System on Pregnancy Outcome of Pregnant Women | Wrong outcome. No weight monitoring |
| Moulaei 2021 | The Development and Usability Assessment of an mHealth Application to Encourage Self-Care in Pregnant Women against COVID-19 | Wrong outcome. No weight monitoring |
| NI 2023 | Efficacy of a Novel Digital Platform to Scale-Up a Personalized Prenatal Weight Gain Intervention Using Control Systems Methodology | Wrong publication type |
| NI 2023 | Increasing Physical Activity Through a Mobile Health Intervention Among Pregnant and Postpartum Women in a Rural Setting | Wrong publication type |
| NI 2023 | Mobile Instant Messaging-based Lifestyle Interventions for Preventing Gestational Diabetes in High-risk Pregnant Women: a Randomised Controlled Trial | Wrong publication type |
| NI 2024 | The Effects of Mobile Health Based Lifestyle Intervention on Gestational Weight Gain in Pregnant Women With Overweight and Obesity: A Randomized Controlled Trial | Wrong publication type |
| NI 2024 | Development and Testing of a Theory-Based Mobile Health App for Weight Management in Pregnancy: A Randomized Controlled Trial | Wrong publication type |
| NI 2024 | Continuous Glucose and E-Monitoring to Support Healthy Weight Gain in Pregnancies - The GEM Study | Wrong publication type |
| NI 2024 | The Impact of Mobile Application-guided Physical Exercise in Healthy Pregnant Women on Vital Physiological Parameters Measured with a Wearable Tracking Device | Wrong publication type |
| NI 2025 | Body Weight Awareness and SUpport for Mums During Pregnancy (BUMP2.0): a Feasibility Study | Wrong publication type |
| NI 2025 | Investigation of the Effectiveness of Social Media-Based Support in Developing Healthy Lifestyle Behaviors in Pregnancy | Wrong publication type |
| NI 2025 | The Effectiveness of Mobile Application Prototype (NutriDent4Mom) in Improving Nutrient Intake, Diet Quality, and Periodontal Health Status of Pregnant Women: A Cluster Randomized Trial | Wrong publication type |
| NI 2025 | Effectiveness of a Smartphone App (PRIMI) to Promote Healthy Diet and Physical Activity After Pregnancy in Migrant Women | Wrong publication type |
| NI 2026 | Effects of an AI-driven Mobile Health Management Intervention to Prevent Gestational Diabetes Mellitus in High-risk Pregnant Women: A Pragmatic Randomized Controlled Trial | Wrong publication type |
| NI 2026 | e-IMPACT: Evaluating an eHealth and Healthcare Professional Support Program to Promote Physical Activity During Pregnancy | Wrong publication type |
| Nicholson 2016 | The Gestational Diabetes Management System (GooDMomS): development, feasibility and lessons learned from a patient-informed, web-based pregnancy and postpartum lifestyle intervention | Wrong population. 100% population with metabolic disease |
| Nicoletti 2026 | Digital Acceptance and Commitment Therapy for Lifestyle Change in Overweight Pregnant Women: A Feasibility Pilot Study | Wrong study design |
| O'Brien 2016 | Delivery of Dietary and Lifestyle Interventions in Pregnancy: is it Time to Promote the Use of Electronic and Mobile Health Technologies? | Wrong study design |
| Ordas 2023 | IUNS 22nd International Congress of Nutrition – Abstracts | Wrong publication type |
| Pollak 2014 | Weight-related SMS texts promoting appropriate pregnancy weight gain: A pilot study | Wrong comparator |
| Potzel 2021 | A Smartphone App (TRIANGLE) to Change Cardiometabolic Risk Behaviors in Women Following Gestational Diabetes Mellitus: Intervention Mapping Approach | Wrong population. 100% population with metabolic disease |
| Rasekaba 2018 | Using technology to support care in gestational diabetes mellitus: Quantitative outcomes of an exploratory randomized control trial of adjunct telemedicine for gestational diabetes mellitus (TeleGDM) | Wrong population. 100% population with metabolic disease |
| Read 2022 | Self-Care Mobile Application for South Korean Pregnant Women at Work: Development and Usability Study | Wrong study design |
| Redman 2017 | Effectiveness of SmartMoms, a Novel eHealth Intervention for Management of Gestational Weight Gain: Randomized Controlled Pilot Trial | Wrong population. 100% population with metabolic disease |
| Roesler 2026 | Preventing Excessive Gestational Weight Gain During Consecutive Pregnancies: Findings from an mHealth Intervention | Wrong study design |
| Roll 2020 | The Feasibility and Preliminary Efficacy of an eHealth Lifestyle Program in Women with Recent Gestational Diabetes Mellitus: A Pilot Study | Wrong population. 100% population with metabolic disease |
| Sandborg 2021 | Participants' Engagement and Satisfaction with a Smartphone App Intended to Support Healthy Weight Gain, Diet, and Physical Activity During Pregnancy: Qualitative Study Within the HealthyMoms Trial | Not available in its full version |
| Seely 2020 | Development and Feasibility of an App to Decrease Risk Factors for Type 2 Diabetes in Hispanic Women with Recent Gestational Diabetes (Hola Bebe, Adiós Diabetes): Pilot Pre-Post Study | Wrong population. 100% population with metabolic disease |
| Seo 2020 | Using a Mobile-based Nutritional Intervention Application Improves Glycemic Control but Reduces the Intake of Some Nutrients in Patients with Gestational Diabetes Mellitus: A Case Series Study | Wrong population. 100% population with metabolic disease |
| Shanmugavel 2024 | Designing and Developing a Mobile App for Management and Treatment of Gestational Diabetes in Nepal: User-Centered Design Study | Wrong study design |
| Sherry 2018 | Inflammation and metabolic health in pregnant women with overweight and obesity, and the impact of a lifestyle intervention | Not available in its full version |
| Simmons 2026 | A randomized digital behavioral intervention for prenatal and postpartum weight outcomes in women with overweight or obesity: the GROWell trial | Wrong comparator. No standard or usual care |
| Téoule 2024 | The effectiveness of integrated online health‑coaching on physical activity and excessive gestational weight gain: a prospective randomized‑controlled trial | Wrong comparator. Comparison included standar care + app |
| Thomas 2018 | A Web-Based MHealth Intervention with Telephone Support to Increase Physical Activity Among Pregnant Patients with Overweight or Obesity: Feasibility Randomized Controlled Trial | Wrong intervention |
| Tian 2021 | Comparing the Efficacies of Telemedicine and Standard Prenatal Care on Blood Glucose Control in Women with Gestational Diabetes Mellitus: Randomized Controlled Trial | Wrong population. 100% population with metabolic disease |
| Tikmani 2024 | Exploring gestational age, and birth weight assessment in Thatta district, Sindh, Pakistan: Healthcare providers’ knowledge, practices, perceived barriers, and the potential of a mobile app for identifying preterm and low birth weigh | Wrong study design |
| Van Dijk 2020 | A mobile app lifestyle intervention to improve healthy nutrition in women before and during early pregnancy: Single center randomized controlled trial | Wrong population. The population includes non-pregnant women |
| Ward 2023 | Feasibility and Acceptability of a Mindfulness-Based Smartphone App among Pregnant Women with Obesity | Wrong outcome |
| Willcox 2020 | Evaluating Engagement in a Digital and Dietetic Intervention Promoting Healthy Weight Gain in Pregnancy: Mixed Methods Study | Wrong study design |
| Woo 2021 | Text Messages to Curb Sugar-Sweetened Beverage Consumption among Pregnant Women and Mothers: A Mobile Health Randomized Controlled Trial | Wrong population. Part of the population are postpartum women |
| Yew 2021 | A Randomized Controlled Trial to Evaluate the Effects of a Smartphone Application-Based Lifestyle Coaching Program on Gestational Weight Gain, Glycemic Control, and Maternal and Neonatal Outcomes in Women with Gestational Diabetes Mellitus: The SMART-GDM Study | Wrong population. 100% population with metabolic disease |
| Yu 2023 | Quality assessment of pre- and postnatal nutrition and exercise mobile applications in the United States and China | Wrong study design |
| Zhang 2019 | Effectiveness of Low Glycemic Index Diet Consultations Through a Diet Glycemic Assessment App Tool on Maternal and Neonatal Insulin Resistance: A Randomized Controlled Trial | No weight monitoring |
| Zhou 2020 | The Effects on Inappropriate Weight for Gestational Age of an SMS Based Educational Intervention for Pregnant Women in Xi'an China: A Quasi-Randomized Controlled Trial | Wrong study design |
| Zhou 2025 | Effects of a Mobile Health Intervention Based on Behavioral Integrated Model on Cognitive and Behavioral Changes in Gestational Weight Management: Randomized Controlled Trial | Wrong outcome |

*NI: not identified*
